# Obsessive trajectories in children and adolescents exposed to adverse events (coronavirus disease 2019: global crisis teaches)

**DOI:** 10.3389/fpsyg.2025.1623629

**Published:** 2025-09-29

**Authors:** Francesco Demaria, Maria Pontillo, Ilaria Bertoncini, Stefano Vicari

**Affiliations:** ^1^Child and Adolescent Neuropsychiatry Unit, Bambino Gesù Children’s Hospital, IRCCS, Rome, Italy; ^2^Department of Life Sciences and Public Health, Catholic University, Rome, Italy

**Keywords:** adverse events, trauma, obsessive-compulsive symptoms/trajectories, automatic intrusive thoughts, children and adolescents

## Abstract

Adverse events (AEs), such as natural disasters, community violence and public health crises, impact global health and are associated with fear, anxiety and disorientation. AEs are related to both short-term and long-term mental health problems in children and adolescents. Particularly, research has shown a significantly higher prevalence of obsessive-compulsive disorder (OCD) in individuals with a history of trauma. This work aims to explore the obsessive-compulsive (OC) trajectories following an AE, considering the role played by individual vulnerability, anxiety and psychological consequences for children and adolescents. In this direction, Coronavirus Disease 2019 (COVID-19) pandemic has represented an ideal and unique AE of concomitant factors that can help to understand the obsessive trajectory. Our framework shows that intrusive flashbacks, following a traumatic experience, can turn into automatic intrusive thoughts that become persistent and emotionally intense, similar to obsessive reactions. Intrusive thoughts can evolve into obsessive patterns, leading to compulsive behaviors aimed at reducing discomfort. The nature of the traumatic event may influence the development of specific OC symptoms. Risk factors include individual vulnerability, such as developmental stage and emotional reactivity, which can exacerbate obsessive stress responses. Anxiety plays a key role, as increased stress can stimulate automatic intrusive thoughts and amplify OCD reactions, especially in younger individuals. Disruptions in daily life can further increase anxiety and maladaptive behaviors in children and adolescents, affecting psychological well-being. The psychological effects of AEs can continue well beyond the events themselves. It is necessary to monitor and support young people involved to prevent their development. Community and individual resources are essential to promote resilience following such events.

## 1 Introduction

Adverse events pose critical challenges to global health. Such events encompass a broad spectrum of phenomena, ranging from natural disasters (e.g., hurricanes, floods, earthquakes) to community violence (e.g., military conflict, terrorism, massacres) and public health crises, including epidemics (e.g., SARS, H1N1, Ebola, COVID-19) elicit widespread fear, anxiety and disorientation within the affected populations (Chrisman and Dougherty, 2014; Maboloc, 2024; Mulenga-Cilundika et al., 2022).

As significant psychosocial stressors, AEs may markedly impact individuals’ lives, with children and adolescents (and their families) standing at particular risk of short- and long-term adverse mental health outcomes (Lai et al., 2017; Sprang and Silman, 2013).

Specifically, elevated stress or anxiety during an AE has been shown to exacerbate OC symptoms in children and adolescents. Notably, individuals with a history of trauma exhibit a significantly higher prevalence of OCD (Palacio-Ortiz et al., 2020).

The present study aimed at examining the potential trajectories of OC symptomatology in children and adolescents following exposure to potentially traumatic events. The investigation considered the interplay of environmental factors associated with AEs, individual vulnerability and anxiety, as well as the broader psychological consequences stemming from the traumatic experience.

The term “OC symptoms” is used to refer to subclinical symptoms that do not significantly interfere with daily functioning or developmental tasks, while “OCD” is used to denote a clinically diagnosed disorder characterized by persistent behavioral and psychological patterns that disrupt functioning and are typically accompanied by anxiety and avoidance behaviors, as outlined in the DSM-5-TR (American Psychiatric Association, 2022).

## 2 Methodology

The study comprised a review of the relevant literature, in accordance with the PRISMA (Preferred Reporting Items for Systematic Reviews and Meta-Analyses) guidelines (Page et al., 2021). An in-depth analysis of academic works was conducted, and sources were carefully verified using electronic databases such as PubMed, CINAHL, PsycInfo, MedLine and the Cochrane Library. Keywords used in the search included: (“Adverse events”) AND (“Trauma”) AND (“Obsessive-compulsive symptoms/trajectories” OR “Automatic intrusive thoughts”) AND (“Children/teenagers”). The review was conducted on March 25, 2025 at 10:30 am, focusing on publications from the preceding 20 years. The inclusion criteria defined original research articles, observational articles (cross-sectional or retrospective) and theoretical or experimental works with psychosocial relevance, addressing pediatric populations during the Coronarovirus Disease 2019 (COVID-19) pandemic. Articles unrelated to the study objectives, duplicates across databases, case reports or case series, and studies aimed at instrument validation were excluded. No restrictions were applied regarding language or study design.

### 2.1 Trauma and OC symptoms: a clinical overlap

The clinical overlap between trauma and OCD is well-documented (Dykshoorn, 2014; Pinciotti et al., 2022), with both conditions characterized by intrusive thoughts, neutralizing behaviors and maladaptive reactions. Traumatic experiences often lead to intrusive symptoms such as flashbacks, hyperarousal, nightmares and irritability. These symptoms are episodic in nature, typically triggered by traumatic memories and marked by intense sensory recall. Moreover, the cognitive impact of trauma is significant: it can impair memory, reduce attentional capacity, contribute to the development of distorted thoughts and beliefs, and hinder the formation of a coherent and integrated memory of the traumatic experience. In contrast, OC symptoms are typically triggered by sudden, emotionally charged experiences perceived as threatening, and frequently manifest as repetitive, intrusive thoughts that are egodystonic. The cognitive profile of OCD often includes marked intolerance of uncertainty, perfectionism, an exaggerated sense of responsibility, overestimation of danger and difficulties with executive functioning (e.g., planning, organization, behavioral regulation) and memory. Under both conditions, children and adolescents may attempt to alleviate distress through compulsive neutralizing behaviors and/or avoidance strategies.

In the initial phase following a traumatic experience, individuals may relive the event through intrusive flashbacks and dysregulated emotional reactions. Over time, these flashbacks can evolve into automatic intrusive thoughts, consolidating the memory of the traumatic event. While these intrusive memories may gradually lose some of their maladaptive intensity, they retain a vivid and emotionally charged nature, acquiring characteristics similar to an “obsessive reaction” and becoming widespread and independent (Fostick et al., 2012; Table 1).

**TABLE 1:** Distinguishing features and overlaps between PTSD-related intrusions and OCD obsessions in children/adolescents.

Symptoms	PTSD intrusion	OCD obsession	Clinical overlaps
Intrusive flashbacks	+	−	−
Intrusive thoughts	−	+	−
Anxiety	+	+	+
Automatic intrusive thoughts	−	−	+
Compulsive neutralizing behaviors	−	+	+
Cognitive impairments	+	+	+
Emotional dysregulation	+	−	+
Avoidance strategies	+	+	+

Sasson et al. (2005) found that the specific attributes of a traumatic event influence the emergence of distinct OC symptomatology.

On a neurobiological level, the Cortico-Striato-Thalamo-Cortical Circuit (CSTC) has been identified as a primary brain system implicated in OCD. The CSTC consists of interconnected neural pathways from the cerebral cortex to the striatum, thalamus, and back to the cortex. It plays a crucial role in several cognitive functions, including motor control, motivation, learning and decision-making. In OCD, two regions within this circuit appear to be significantly implicated: the anterior cingulate cortex (ACC) and the orbitofrontal cortex (OFC), both of which are critical in the emergence and persistence of obsessive symptoms (Dougherty et al., 2018). The OFC is involved in risk assessment, behavioral regulation in response to environmental stimuli and the attribution of meaning to those stimuli. In the context of OCD, this function becomes dysregulated, leading to an overestimation of the negative consequences of a given action, the emergence of intrusive thoughts and a persistent perception of danger. Moreover, the ACC, which governs error monitoring, motivational drive and urgency, contributes to the compulsive dimension of OCD by generating the feeling that something is not “complete” or “correct,” thereby triggering compulsive behaviors. For example, the hygiene-related compulsions (e.g., excessive handwashing) observed among certain youth populations during the pandemic may reflect an environmentally conditioned response aimed at reducing anxiety and fear, underpinned by CSTC activation (Ornell et al., 2021).

At the neurotransmitter level, CSTC dysfunction is associated with reduced serotonergic and GABAergic inhibitory input from both medial prefrontal cortex interneurons and the midbrain raphe nuclei. This deficit leads to hyperactivity in the ACC and OFC, which in turn drives dopaminergic and glutamatergic hyperactivity in the striatum and thalamus, resulting in unchecked CSTC activity (Dougherty et al., 2018; Karthik et al., 2020).

The neurobiology of OCD and post-traumatic stress disorder (PTSD) also demonstrates notable overlap. Both conditions involve dysfunctions in specific brain areas—particularly the amygdala and prefrontal cortex— that are linked via circuits associated with stress and fear. The hyperactivation of these networks contribute to heightened anxiety and the emergence of intrusive thoughts or images. The amygdala, in particular, plays a central role in the consolidation of emotional memory, preserving experiences accompanied by strong emotional intensity and rendering them more lasting and meaningful. Thus, non-rational fear and anxiety may arise from traumatic events that have become fixed in emotional memory (Cheng et al., 2015).

### 2.2 The COVID-19 pandemic as a model of emerging OC symptoms

During the COVID-19 pandemic, Darvishi et al. (2020) documented that washing compulsions were 68 the most common OC symptom in a non-clinical sample of students. The authors found that young 69 people may have developed compulsive hand-washing behaviors after learning that this 70 recommended action helped alleviate their anxiety. These behaviors could be attributed to a fear of 71 contagion and an obsession with hygiene. It is therefore plausible that intrusive thoughts stemming 72 from a traumatic experience may become structured into obsessive patterns, triggering repetitive or 73 compulsive behaviors aimed at reducing the distress caused by such obsessions (Darvishi et al., 2020).

A key risk factor for the emergence of OC symptoms following an AE is individual vulnerability. 75 Secer and Ulas demonstrated that adverse environmental conditions (e.g., fear of contagion 76 during the COVID-19 pandemic), combined with subjective vulnerabilities (e.g., heightened 77 emotional reactivity, avoidance behaviors, anxiety-depression), can act as precipitating factors for 78 OC symptoms (Seçer and Ulaş, 2021). Similarly, Raymond et al. (2022) found that post-traumatic stress symptoms and 79 anxiety were common among young people with socio-emotional weakness, characterized by 80 anxious traits, intolerance to uncertainty, and a tendency toward rumination.

Adult studies, particularly those involving civilians and small samples of war veterans, have long documented the presence of OCD in individuals with a history of trauma (Kessler et al., 2005). For instance, Morina et al. (2016) studied a sample of 51 adult civilians (28 males) who survived the Kosovo War, finding that 35 and 39% scored above the clinical threshold for probable OCD and PTSD, respectively. Notably, participants exhibiting elevated PTSD symptoms were significantly more likely to present with OC symptoms, and vice versa (Morina et al., 2016).

In contrast, the scientific literature on pediatric populations remains comparatively limited. Nonetheless, understanding OCD symptom onset and development in children and adolescents exposed to trauma is crucial for elucidating the relationship between trauma and compulsive symptomatology.

The unique and large-scale AE of the COVID-19 pandemic offers a valuable context for examining the emergence of OCD symptom trajectories, particularly in pediatric populations. The pandemic simultaneously introduced multiple and varied stressors, including fear of contagion, stringent protection measures, environmental disruption, personal trauma and individual vulnerability. Moreover, different from isolated AEs (e.g., natural disasters, localized community crises), the pandemic was characterized by its global scale, prolonged nature and chronic uncertainty. It also featured significant social isolation, media saturation and environmental and familial stress.

Review articles and meta-analyses have documented these adverse effects (Ma et al., 2021) in children and adolescents.

Cunning and Hodes (2022) demonstrated that certain factors related to the pandemic (e.g., lockdown measures, heightened attention to hygiene, family-related adversities that intensified fear of contagion) were associated with increased OCD symptoms. Particular emphasis was placed on hygiene-related behaviors (e.g., handwashing) and contamination fears, as well as the role played by the media, which perpetuated anxiety through continuous updates on virus transmission, mortality rates and quarantine protocols. These factors collectively heightened the perceived threat among adolescents during the pandemic (Liu et al., 2020).

It is plausible that certain groups of adolescents possess a lower threshold for tolerating AEs, rendering them more susceptible to developing OC symptoms as a maladaptive coping mechanism (Chen et al., 2020). Supporting this hypothesis, Seçer and Ulaş (2021), in a study involving 598 high school students, found that adverse environmental conditions (e.g., fear of contagion during the pandemic), when combined with individual vulnerabilities (e.g., heightened emotional reactivity, avoidance behaviors, anxiety-depressive symptoms) significantly predicted the emergence of OCD symptoms. In particular, emotional reactivity and experiential avoidance were identified as key risk factors for broader psychosocial disturbance. These findings align with those of Raymond et al. (2022) who observed that anxiety and PTSD symptoms were prevalent among adolescents with socio-emotional vulnerabilities during the pandemic, characterized by anxious traits, intolerance of uncertainty and a tendency toward rumination. Consistently, anxiety has been highlighted as a critical dimension of individual vulnerability in the development of OC symptoms in young populations (Rueppel et al., 2024).

### 2.3 The role of anxiety and intolerance of uncertainty

Vulnerability to anxiety may constitute a significant risk factor in the development of OC symptoms. Anxiety and OCD share several clinical and behavioral features, including persistent rumination, excessive worry and avoidance behaviors. During the COVID-19 pandemic, the emotional burden— driven by an unfamiliar virus, absence of definitive treatment and potential lethality—was particularly intense, often leading to overwhelming anxiety and worry. These factors may have contributed to intrusive thoughts related to contagion and disease, which, in some cases, may have triggered OC symptomatology. Darvishi et al. (2020) investigated this phenomenon in a non-clinical sample of students during the pandemic, reporting that handwashing compulsions were the most commonly observed OC symptom. The authors further proposed that young people may have developed compulsive handwashing behaviors as a response to the relief they experienced after engaging in a recommended public health practice.

Intolerance of uncertainty has also been identified as a key factor in both anxiety and OC symptoms, particularly in high-risk or ambiguous situations (Gillett et al., 2018). This dynamic was particularly evident during the early stages of the COVID-19 pandemic, when unprecedented levels of uncertainty, coupled with lockdowns and stringent protective measures, created an environment that exacerbated OC symptoms. Data collected during March and April 2020 consistently supported this observation, demonstrating a sharp rise in OC symptomatology linked to the unique challenges of the pandemic (Singh et al., 2020).

**FIGURE 1:**
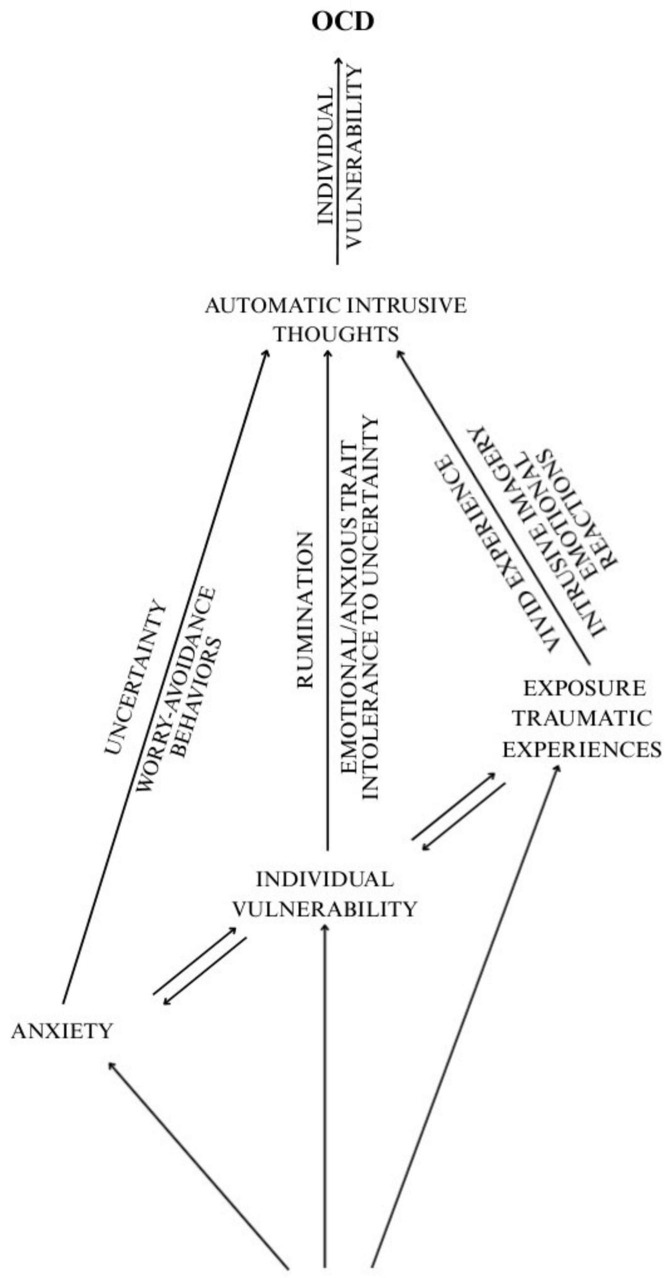
Obsessive child/adolescentb trajectory in AE.

It is difficult to understand the precise nature of obsessive thinking and its manifestations across varying emotional states, individual predispositions and environmental conditions. However, it is evident that children with specific vulnerabilities, when exposed to prolonged stress, persistent uncertainty and intense environmental stimuli, are more likely to develop repetitive thought patterns that may evolve into intrusive thoughts and, ultimately, obsessions. In individuals experiencing intense anxiety, obsessions are typically accompanied by heightened uncertainty, worry and avoidance behaviors. Among those with distinct socio-emotional vulnerabilities (e.g., anxious temperament, ruminative tendencies, intolerance to uncertainty), obsessive thinking may be further reinforced. In cases involving traumatic exposure, vivid subjective experiences and intrusive mental imagery (e.g., flashbacks of emotionally salient memories) often play a central role in activating and sustaining obsessive thoughts (Figure 1).

This body of evidence underscores the heightened psychological vulnerability of children and adolescents during AEs and the role of neurobiological plasticity in shaping responses to environmental stimuli.

## 3 Discussion

The impact of AEs on the lives of young individuals extends beyond their immediate emotional state, often inducing broader environmental, familial, and community stress. For instance, during the COVID-19 pandemic, lockdowns and necessary quarantine measures led to substantial lifestyle changes, accompanied by heightened anxiety, fear of infection, and pervasive concern. These dynamics compromised individuals’ ability to implement effective coping strategies, and the limited availability of psychiatric support services further exacerbated the situation (World Health Organization, 2020).

Understanding the relationship between AEs and the onset of OCD is crucial for advancing knowledge of its pathogenesis. Approximately 50% of adults with OCD report an early onset of symptoms before the age of 18 years (Geller et al., 2021). AEs and traumatic experiences may act as catalysts for obsessive trajectories, influencing clinical symptomatology and complicating long-term recovery. In children and adolescents, the obsessive consequences of AEs often persist long after the stressful event, exerting significant and enduring effects on their psychological well-being.

A cross-sectional survey (Habib and Zulfiqar, 2025) investigated the prevalence and comorbidity of PTSD and post-traumatic OCD among Afghan refugees in Pakistan, with a focus on the intergenerational transmission of PTSD. The study involved 48 male adults representing three generations of 16 refugee families. The results revealed a significant correlation between post-traumatic OCD and PTSD, indicating a substantial co-occurrence between the two disorders. Overall, 79.20% of participants received a diagnosis of post-traumatic OCD, with higher prevalence in the first generation (87.5%) compared to the second and third generations (75.0%). Additionally, PTSD prevalence varied significantly across generations, with a trend toward reduced symptom severity over time. These findings underline the need to address the comorbidity of both disorders in populations exposed to AEs, while also considering intergenerational negative outcomes in terms of mental health.

It is therefore imperative to closely monitor young individuals and provide continuous support to prevent the long-term development of psychological disorders. Lowe et al. (2015), in their study on psychological distress following Hurricane Sandy, emphasized the importance of leveraging both community and individual resources to foster resilience in post-disaster contexts.

### 3.1 Potential confounders and limitations of the reviewed studies

Many of the studies examined (most of which were conducted during the pandemic) were cross-sectional in nature, relying on self-report questionnaires or online surveys. Such methodologies limit the ability to assess formal psychiatric diagnoses or pre-existing subclinical conditions. As a result, the reported prevalence rates may be inflated or include false positives, particularly when the studies failed to account for participants with genetic predispositions to OCD or those from socio-economically disadvantaged backgrounds—both of which may increase vulnerability to psychopathology following AEs. AEs tend to disproportionately affect economically and socially marginalized populations, with chronic stress, limited access to care and overcrowded living conditions acting as potential confounding factors that may intensify anxiety and compulsive symptoms. Finally, accurate differentiation between normative and pathological behavior requires diagnoses in clinical settings, posing a further methodological limitation.

### 3.2 Limitations

The review itself is subject to several limitations. First, the data examined were predominantly derived from studies focused on the COVID-19 pandemic, potentially limiting the generalizability of the findings to other types of AEs and reducing analytical depth. Second, there was a lack of neurobiological evidence to support the correlation between AEs and OC manifestation in children and adolescents. Additionally, the lack of longitudinal studies of youth prevented a comprehensive and robust evaluation. Finally, the narrative and non-systematic nature of the review inherently constrained the scope and reliability of its conclusions.

## 4 Conclusion

In conclusion, adverse events (AEs) AES significantly impact the mental health of children and adolescents, often correlating with the development of OC symptoms. The interplay between trauma, individual vulnerability, and environmental stressors plays a crucial role in shaping the trajectory of these symptoms. The COVID-19 pandemic highlighted how environmental changes, such as fear and uncertainty, can exacerbate OC behaviors, especially in vulnerable youth.

It is essential that mental health services for children and adolescents ensure adequate and timely psychological and/or psychiatric support during exceptional circumstances (e.g., lockdowns, social restrictions). Such support should be coordinated with social welfare services and, more broadly, with local and national civil protection services. In crisis contexts, diverse and alternative intervention strategies must be considered. Remote modalities (e.g., telephone, online) may be necessary to provide psychological or educational assistance for both children and their caregivers. Additionally, innovative approaches aligned with the principles of digital psychiatry, such as those incorporating artificial intelligence and internet-based technologies, should be explored (Ćosić et al., 2020).

Emerging digital technologies hold promise for early screening and prevention among high-risk populations. Local health authorities should distribute accessible family guidelines promoting healthy cohabitation strategies and public health measures, and media outlets should play a central role in promoting awareness and disseminating reliable information. Finally, the psychological well-being of children should be monitored in both the short and long term, particularly among those demonstrating socio-emotional vulnerability. Early identification of obsessive or post-traumatic symptoms—and of trajectories suggestive of OC patterns—may support timely intervention and the strengthening of psychological resilience.

Understanding the links between trauma and OCD is vital for improving therapeutic strategies and fostering resilience in affected individuals.
